# Vitality, fermentation, aroma profile, and digestive tolerance of the newly selected *Lactiplantibacillus plantarum* and *Lacticaseibacillus paracasei* in fermented apple juice

**DOI:** 10.3389/fnut.2022.1045347

**Published:** 2022-12-06

**Authors:** Jia Rui Liang, Hong Deng, Ching Yuan Hu, Peng Tao Zhao, Yong Hong Meng

**Affiliations:** ^1^The Engineering Research Center for High-Valued Utilization of Fruit Resources in Western China, Ministry of Education, National Research & Development Center of Apple Processing Technology, College of Food Engineering and Nutrition Science, Shaanxi Normal University, Xi’an, China; ^2^Department of Human Nutrition, Food and Animal Sciences, College of Tropical Agriculture and Human Resources, University of Hawai’i at Mānoa, Honolulu, HI, United States

**Keywords:** *Lactobacillus*, organic acids, phylogenetic tree, viable count, volatile compounds

## Abstract

**Background:**

To enrich the probiotic lactic acid bacteria (LAB) strains and expand the commercialization of new fermented juice products, we have identified two LAB strains with excellent potential in fermenting apple juice from pickles.

**Methods:**

The two strains were morphologically, physiologically, and genetically characterized. The strains’ fermentation performance and alterations in volatile aroma components of apple juice and ability to survive in a simulated gastrointestinal environment were evaluated.

**Results:**

Two strains were identified as *Lacticaseibacillus paracase*i (WFC 414) and *Lactiplantibacillus plantarum* (WFC 502). The growth of WFC 414 and WFC 502 in apple juice for 48 h reached 8.81 and 9.33 log CFU/mL, respectively. Furthermore, 92% and 95% survival rates were achieved in 2 h simulated gastric juice, and 80.7 and 83.6% survival rates in 4 h simulated intestinal juice. During the fermentation, WFC 414 and WFC 502 reduced the soluble sugars and total polyphenols in apple juice, and consumed malic acid to produce large amounts of lactic acid (3.48 and 5.94 mg/mL). In addition, the esters and aldehydes were reduced, and the production of alcohols, acids and ketones was elevated in the apple juice fermented by both strains.

**Conclusion:**

These results show that WFC 414 and WFC 502 have great potential applications in the fermented fruit juice industry.

## Introduction

Microbial fermentation has been one of the most commonly used food processing and preservation since ancient times (1). With the action of beneficial microorganisms, the sugars, lipids, and proteins in food materials are decomposed and converted into unique flavors and other nutrients (2). As a result, fermented dairy products such as yogurt, milk beverages, and cheeses have been popular in the past few decades. Moreover, as consumers have become more aware of the harms of lactose intolerance and high cholesterol in recent years, fermented juices based on non-dairy products, such as fruits and vegetables, have attracted more attention from consumers and researchers in the food field (3).

Lactic acid bacteria (LAB) produce lactic acid by fermenting carbohydrates in juices. Fermentation improves flavor and texture, enhances nutrition and quality, and extends the shelf life of juices (4). Apple juice, an excellent substrate for LAB fermentation, has valuable nutrition due to its high carbohydrates, polyphenols, vitamins, minerals, and dietary fibers. Moreover, ingesting apples can reduce the risk of diseases and prevent chronic diseases, cancer and cardiovascular diseases due to the bioactive compounds in apples (5). However, the industrial processing of apple juice, such as pasteurization, steaming, and chemical preservatives, is causing undesirable physical, chemical and nutritional changes to the products (6). Fermented apple juice (FAJ) by LAB is one of the most effective and convenient methods to improve this situation. The strains in traditional fermented foods are complex and diverse, rich in nutrition, and more suitable for food fermentation. Compared with natural microorganism fermentation, fruits fermented by commercial LAB isolated from traditional fermented foods may generate a satisfactory flavor, allow standard and safe control of the fermentation process, and stabilize the quality of the product (7). Therefore, screening for more LAB suitable for apple juice fermentation to enrich the field of fermented foods is a worthy challenge.

Among the LAB used in fermenting apples, *Lactobacillus* (8) is one of the most common genera used as a probiotic (4). *Lactobacillus* has been reported to modify the phenolic composition of apple juice and enhance its overall antioxidant capacity (9). Single strain fermentation by *Lactobacillus plantarum* (*L. plantarum*) and *Lactobacillus reuteri* exhibited a higher viable count and better superoxide dismutase (SOD) activity in apple juice (10). *L. plantarum* fermentation also showed increased 2,2-diphenyl-1-picrylhydrazyl (DPPH) and 2,2′-azinobis(3-ethylberizothiazoline-6-sulfonic acid) (ABTS) radical scavenging activity (11). Fermentation of *L. plantarum*, *Lactobacillus helveticus*, *Lactobacillus acidophilus* (*L. acidophilus*), and *Lactobacillus paracasei* (*L. paracasei*) could enrich the background flavors of apple juice by increasing alcohols, ketones, aldehydes, and acids in the volatile aroma components (12, 13). At the same time, fermentation significantly reduces the substances that cause unpleasant tastes. Apple juices fermented by *L. acidophilus*, *Lactobacillus fermentum*, and *L. plantarum* inhibited weight gain, reduced total cholesterol (TC), triglyceride (TG), low-density lipoprotein cholesterol (LDL-C) levels, increased high-density lipoprotein cholesterol (HDL-C) level, maintained the balance of gut microbiota and protected intestinal tract health effectively (14, 15).

Consumers demand improved organoleptic quality, enhanced health properties, and diversified juice products. Therefore, volatile aroma components of fermented juice play significant roles in consumers’ preferences. Simultaneously, many studies suggest that the intake of fermented foods improves gut microbiota and thus modulates immune response and other chronic diseases (16, 17). Therefore, screening for LAB strains with significant aroma enhancement is imperative for fermented juice production and effective colonization in the intestinal tract. However, specific starters with pleasant flavor and strong survival ability for fruit juice fermentation are still under development (15).

In this study, we isolated and screened 80 strains from traditional Chinese pickles, selected and identified two strains, *Lactiplantibacillus plantarum* WFC 502 and *Lacticaseibacillus paracasei* WFC 414. Both strains have high viability and aroma-producing capacity. We compared the survival of two strains in simulated gastric and intestinal conditions and changes in apple juice’s soluble sugar, organic acid and volatile compounds content before and after fermentation. Both strains have excellent acid tolerance and intestinal colonization ability and increased the type and amount of favorable aroma-presenting compounds such as alcohols and ketones in the juice. In addition, *L. plantarum* WFC 502 has a stronger ability to metabolize carbohydrates. The novelty of this study is the development of two strains isolated from traditional Chinese fermented foods that have excellent potential to be used in apple juice fermentation. They enrich the field of novel fermented foods and provide more options for commercializing healthy and nutritious apple juice products.

## Materials and methods

### Preparation of apple juice

Fresh Fuji apples (purchased from an apple orchard in Changwu county, Shaanxi, China) without pests and diseases were peeled, cored, cut in half and immediately soaked in 0.5% ascorbic acid solution for 15 s to suppress enzymatic browning. Subsequently, the apple pulps were crushed by a juice extractor (HUF8800STS, HUROM; Seoul, South Korea) and filtered through a double gauze. The juice was then heated from room temperature to 90°C and boil slightly for 20 s as pasteurization. Finally, the juice was canned while it was still hot, cooled naturally, and stored at 4°C.

### Isolation and preparation of strains

The four types of pickles purchased were radish (Jinzhou, Liaoning, China), cabbage (Xinmin, Liaoning, China), sauerkraut (Suining, Sichuan, China), and a mixture of dandelion, cabbage, and celery (Tianshui, Gansu, China). The 0.1 mL of each pickle juice was aspirated, diluted and applied to MRS agar medium and incubated under the anaerobic condition at 37°C for 48 h. Afterward, 20 single colonies from different pickle plates were picked and purified by streaking on MRS agar. Purified strains were cultured in MRS broth at 37°C in an incubator (ZC-250 thermostatic oscillator, PeiYing experimental equipment Co.; Suzhou, China) at 100 rpm for 24 h with anaerobic bags.

### Fermentation of apple juice

The bacteria cultured in MRS broth were centrifuged at 5,000 × *g* for 5 min. Then, the obtained bacterial cells were washed twice with physiological saline and resuspended in an equal volume of physiological saline. The resuspension was inoculated into pasteurized apple juice (PAJ) at 1% (v/v) inoculum to achieve a viable count of about 6 log CFU/mL in the juice prior to fermentation. Fermentation was performed in triplicate and anaerobically in an incubator at 130 rpm at 37°C for 48 h.

### Screening of strains

The FAJ was sniffed to remove strains that produced distinctly unpleasant odors and less inconspicuous aromas during fermentation. Then, the viable cells were counted before and after fermenting the apple juice by the standard serial dilution method with sterile physiological saline. The diluted FAJ samples were spread on MRS agar plates and incubated inverted at 37°C for 48 h in an incubator. The strains with the largest and fastest increase in the number of viable cells during fermentation were selected. Meanwhile, the FAJ was diluted and coated in MRS agar plates supplemented with 0.7% CaCO_3_, and incubated inverted at 37°C for 48 h to observe whether a calcium-dissolving circle was formed. The strains that produced a calcium-dissolving circle were considered to be LAB.

Ten trained candidates (five males and five females) evaluated the sensory properties of FAJ for five aroma descriptors (fruity, sweet, grassy, floral, and pungent). A nine-point scale (0 = none, 9 = exceptionally strong) was used for scoring and a spider web diagram was drawn (18).

### Identification of the selected lactic acid bacteria isolates

Including catalase activity, gelatin liquefaction, indole, and hydrogen sulfide tests, were performed following the published methods (19).

For sugar fermentation tests: Each LAB isolate was inoculated with arabinose, cellobiose, hesperidin, mannitol, fructose, honey disaccharide, cottonseed sugar, salicin, galactose, glucose, maltose, sucrose, and alginate using a micro-identification biochemical tube (SHBG13, Hope Bio-Technology; Qingdao, China) and incubated at 37°C for 24 h, respectively. The change from purple to yellow in the biochemical tube indicates the generation of acid, which is a positive reaction.

The 16S rRNA of *Lactobacillus* screened from pickles were extracted, amplified and sequenced, and then compared with the sequences stored on BQ GenBank-EMBL by the BLAST program^1^ of the National Center for Biotechnology Information (NCBI), and identified to a species with % of homology greater than 97%. Next, the sequences of different *LAB* strains reported in the relevant studies were selected, and the phylogenetic tree was drawn using MEGA X 10.0.2 software.

### Survival of *Lactobacillus* exposed to simulated gastrointestinal conditions

The simulated gastric juice (SGJ) and simulated intestinal juice (SIJ) were prepared according to Moayyedi et al. (20). One g of *Lactobacillus* cells was inoculated into nine mL of sterile SGJ and SIJ solution and incubated at 37°C for 2 and 4 h with shaking at 50 rpm, respectively. Afterward, the samples were counted on MRS agar, and the survival rates were calculated.

### Measurement of soluble sugars and organic acids

Soluble sugars (fructose, glucose, and sucrose) were analyzed using High-Performance Liquid Chromatography (HPLC 1260 Infinity, Agilent Technologies, CA, USA) according to methods slightly modified from the National Standards of the People’s Republic of China (GB 5009.8-2016). Briefly, 1 mL of each sample was added to the injection bottle and placed on the autosampler of the chromatograph equipped with a ZORBAX NH_2_ column (4.6 × 250 mm, 5 μm; Agilent) and a refractive index detector. The mobile phase was acetonitrile: water (70:30) at a 1.0 mL/min flow rate. The injection volume was 10 μL. The temperature of the column and detector was kept at 30°C.

Organic acids (malic acid, lactic acid, acetic acid, oxalic acid, pyruvic acid, and citric acid) were analyzed using HPLC according to Li et al. (21). Briefly, 1 mL of each sample was added to the injection bottle and placed on the autosampler of the chromatograph equipped with a TC-C18 column (250 × 4.6 mm, 5 μm; Agilent) and an ultraviolet detector. The mobile phase was 0.01 mol/L KH_2_PO_4_ (adjusted to pH 2.7 using H_3_PO_4_): methanol (97:3) at a 0.7 mL/min flow rate, with an injection volume of 10 μL. The column temperature was maintained at 25°C, and the wavelength of the ultraviolet detector was 210 nm.

The PAJ and FAJ samples were centrifuged, and the supernatant was filtered through a 0.22 μm membrane (polyethersulfone filter). Identification and quantification were performed based on standard solutions’ retention times and calibration curves.

### Measurement of total polyphenols and individual phenolic compounds

Total polyphenol content was measured according to the method of Wu et al. (13) with modifications. One mL of juice and 2.5 mL of Folin-Ciocalteu were mixed and reacted for 3 min, then 2.5 mL of 15% Na_2_CO_3_ was added to the mixture and vortexed for 1 min. Subsequently, the mixture was reacted for 60 min at room temperature in the dark and the absorbance was measured at 765 nm. The total polyphenol content was expressed as gallic acid equivalent (mg GAE/L).

The juice was mixed with 80% methanol solution in the ratio of 1:2 and the polyphenols were extracted by sonication at 25°C for 15 min, then centrifuged at 8,000 rpm for 10 min at 4°C. The supernatant was then filtered through a 0.22 μm membrane (polyethersulfone filter). The content of individual phenolic compounds was determined using HPLC equipped with an ultraviolet detector and a TC-C18 column according to the method of Tian et al. (22). The mobile phase A (30% acetonitrile and 70% methanol) and mobile phase B (1‰ trifluoroacetic acid and 5% methanol) were eluted in a gradient as follows: 0–3 min, 100% B; 3–19 min, 100–60% B; 19–30 min, 60% B; 30–31 min, 60–100% B; 31–41 min, 100% B. The flow rate was 1 ml/min and the wavelength was 289 nm, and the wavelength of ultraviolet detector was 280 nm.

### Determination of volatile compounds

The volatile compounds in PAJ and FAJ samples were determined by a Headspace Solid-Phase Microextraction coupled with Gas Chromatography-Mass Spectrometer (HS-SPME-GC/MS) method described by Yang et al. (23) with certain modifications.

### Headspace-solid-phase microextraction

Five mL of sample and 2 g NaCl were mixed in a 20 mL headspace vial with 10 μL 3-octanol (100 mg/L in methanol) as the internal standard. The vial was stirred at 60 rpm in a 50°C water bath (DF-101S; DUFU YIQI; Zhengzhou, China) for 30 min. A manual SPME fiber sampler (50/30 μm, DVB/CAR/PDMS, 2 cm, Supelco; Bellefonte, PA, USA) was used for extraction. The fiber was exposed to the upper gas of the headspace vial and continued to stir at 50°C for 30 min. Afterward, the sampler was instantly inserted into the GC injector for thermal desorption at 250°C for 3 min.

#### Gas chromatography-mass spectrometry analysis

The volatiles were separated by GC/MS (QP2010SE, Shimadzu, Japan) instrument equipped with a DB-wax capillary column (30 m × 0.25 mm i.d., 0.25 μm film thickness). Helium (99.999%) was used as the carrier gas at a 3 mL/min flow rate. The gas chromatograph temperature was increased from 40°C (held for 3 min) to 100°C (held for 5 min) at a rate of 10°C/min and then to 230°C (held for 10 min) at a rate of 4°C/min. The mass spectrum was scanned by electron ionization (EI) mode at 70 eV to obtain a range of 35–500 m/z.

The concentration of each compound is a relative concentration calculated based on the concentration of the internal standard 3-octanol added [Eq. (1)].


(1) $$N_{x}=\frac{S_{x}\times N_{O}}{S_{O}}$$

where N_x_ and N_O_ are the compounds and 3-octanol concentration (μg/L), respectively; S_x_ and S_O_ are the GC peak areas of the compounds and 3-octanol, respectively.

### Statistical analysis

All experiments were conducted in triplicate, and the results are given as means ± standard deviation. Statistical data were evaluated using ANOVA, and the comparison tests were verified by Duncan’s test using SPSS 20.0 (SPSS Inc.; Chicago, IL, USA). A value of *p* < 0.05 was considered statistical significance. Column charts and line graphs were plotted using Origin 2021 software (OriginLab, Northampton, MA, USA).

## Results and discussion

### Lactic acid bacteria isolated from traditional pickles

**FIGURE 1:**
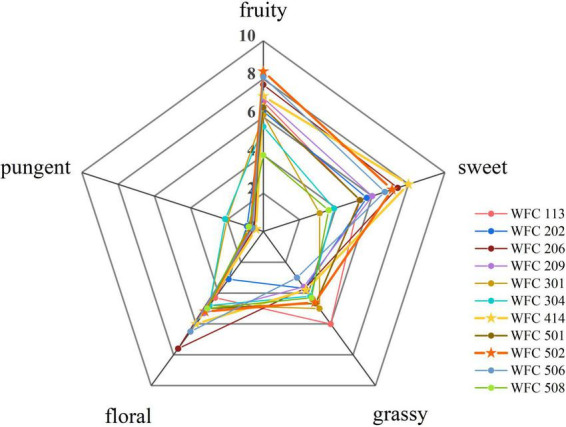
Sensory profiles of apple juice fermented by different strains.

Lowering the pH through LAB fermentation improves the antibacterial activity of the juice and provides a soft sour taste (24, 25). Besides, the aroma of the fermented fruit juice has a crucial influence on the character and marketability of the final product (26, 27). Therefore, the LAB from the pickles was isolated to select the strains with strong aroma production ability, high viable cell counts, and noticeable pH reduction in fermenting apple juice. Their aroma intensity, viable cells, and pH of the fermented apple juice were determined. A total of 80 strains of bacteria were isolated from four types of pickles purchased, and each strain was numbered. First, the odor of fermented apple juice for each strain was sniffed, in which 20 strains with an unpleasant odor, 17 strains with no apparent acceptable aroma, 32 strains with light aroma, and 11 strains with strong aroma after fermentation were identified. Then, a secondary screening was conducted for these 13 strains (Table 1 and Figure 1). WFC 414 and WFC 502 strains showed significant pH drop, the highest number of viable cells (8.81 ± 0.13 and 9.33 ± 0.12 log CFU/mL), the strongest aroma after fermentation, and a calcium soluble circle were selected. The properties and fermentation characteristics of these two strains were further studied in the later experiments.

**TABLE 1:** Fermentative profiles of apple juice by the different strains isolated in the second screening session.

	WFC 113	WFC 202	WFC 206	WFC 209	WFC 301	WFC 304	WFC 414	WFC 501	WFC 502	WFC 506	WFC 508
Initial pH	4.90 ± 0.09	4.90 ± 0.09	4.90 ± 0.09	4.90 ± 0.09	4.90 ± 0.09	4.90 ± 0.09	**4.90 ± 0.09**	4.90 ± 0.09	**4.90 ± 0.09**	4.90 ± 0.09	4.90 ± 0.09
Final pH	5.19 ± 0.07	4.71 ± 0.07	6.33 ± 0.11	4.14 ± 0.06	4.66 ± 0.10	4.50 ± 0.10	**3.97 ± 0.07**	4.38 ± 0.05	**3.74 ± 0.05**	5.61 ± 0.08	6.91 ± 0.03
Initial viable cells (log cfu/mL)	6.75 ± 0.09	5.77 ± 0.09	5.86 ± 0.03	6.10 ± 0.15	5.91 ± 0.03	5.81 ± 0.04	**5.79 ± 0.16**	5.97 ± 0.08	**6.26 ± 0.04**	5.94 ± 0.07	5.89 ± 0.06
Final viable cells (log cfu/mL)	8.75 ± 0.18	8.62 ± 0.04	7.97 ± 0.03	8.57 ± 0.09	7.58 ± 0.06	7.42 ± 0.11	**8.81 ± 0.13**	8.53 ± 0.14	**9.33 ± 0.12**	8.02 ± 0.09	8.12 ± 0.13
Dissoluble calcium	–	+	–	+	+	–	**+**	+	**+**	–	+
Aroma intensity	++	++	+++	+++	++	+	**+++**	++	**+++**	+++	+

Bold values represent the two strains selected in this study.

### Morphological, physiological, and biochemical characteristics of the selected isolates

**FIGURE 2:**
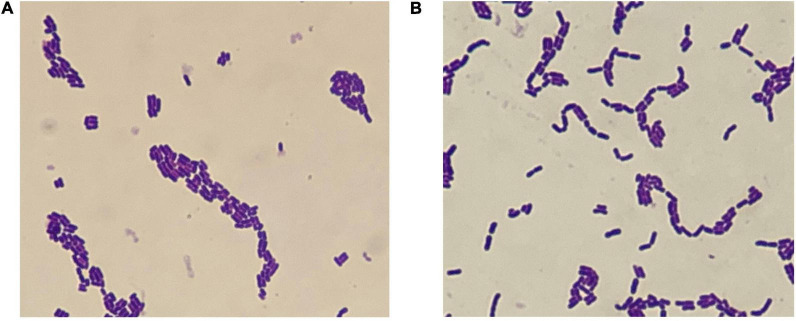
Micromorphology of WFC 414 **(A)** and WFC 502 **(B)**.

The characteristics of WFC 414 and WFC 502 are shown in Figure 2 and Table 2. Microscopic observation reveals that both strains were Gram-positive, non-motile, and non-budding short bacilli. Meanwhile, the two strains’ biochemical test results were the same: peroxidase negative, non-liquefying gelatin, non-indole and non-hydrogen sulfide production. Based on these, WFC 414 and WFC 502 are tentatively identified as *Lactobacillus* spp. Both strains reacted positively with esculin, cellobiose, mannitol, fructose, salicin, galactose, glucose, maltose, sucrose, and trehalose. The WFC 502 fermented melibiose and raffinose, while the WFC 414 could not. The properties of WFC 414 are similar to those of the *Lacticaseibacillus casei* group. *Lacticaseibacillus casei*, *Lacticaseibacillus paracasei*, and *Lacticaseibacillus rhamnosus* belong to the *L. casei* group, and they share many features that lead to especially similar phenotypes (28). While WFC 502 is consistent with *Lactiplantibacillus plantarum*. Although these methods can effectively distinguish strains at the species level, identification at the genetic level is the most adequate for discrimination (29).

**TABLE 2:** Biochemical and sugar fermentation characteristics of WFC 414 and WFC 502.

		WFC 414	WFC 502
Biochemical	Hydrogen peroxidase	–	–
	Gelatin liquefaction	–	–
	Indole	–	–
	Hydrogen sulfide	–	–
Sugar fermentation	Esculin	+	+
	Cellobiose	+	+
	Mannitol	+	+
	Fructose	+	+
	Melibiose	–	+
	Raffinose	–	+
	Salicin	+	+
	Galactose	+	+
	Glucose	+	+
	Maltose	+	+
	Sucrose	+	+
	Trehalose	+	+

+, positive; –, negative.

### Identification and phylogenetic tree of *Lactiplantibacillus plantarum* and *Lacticaseibacillus paracasei*

**FIGURE 3:**
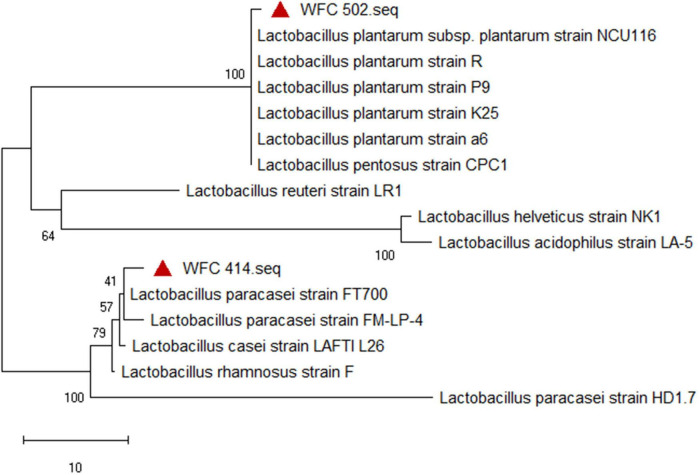
Position of the WFC 414 and WFC 502 strains in the neighbor joining phylogenetic tree.

The micrograph, physiological, and biochemical identification of *Lactobacillus* can only initially determine the similarity of phenotypic characteristics, which does not mean that the genotypic relatives are similar. And the genotypic characteristics need to be further identified. The two strains’ 16S rRNA gene partial fragments were obtained by extracting and sequencing. A comparison of the gene fragments in the NCBI BLASTn revealed that the homology of WFC 414 (accession No. OP735581) with *the L. paracasei* sequence was higher than 98%. And WFC 502 (accession No. OP735580) shared 99% identity with the gene sequence of *L. plantarum*. The phylogenetic tree reconstructed by the neighbor-joining method reveals a phylogenetic relationship between the two strains’ nucleotide sequences of the 16S rRNA gene and other reported LAB strains (Figure 3). The species of the two strains were unambiguously clarified through the phylogenetic tree’s topology and the strains’ relative positions. The isolated strain WFC 414 has closely related genotypes with the phylogenetic clade of *L. paracasei*. *L. paracasei* is often isolated from the intestinal tract and plants and has been widely and commercially used in fermented fruits, beverages, yogurt, and other fermented foods (30–32). In addition, WFC 502 has a 100 bootstrap value with the phylogenetic clade of *L. plantarum*. *L. plantarum* is the most common LAB in fermented fruit juices. It has also been found to have specific effects, such as inhibiting colorectal tumors and alleviating oxidative stress in mice (33). According to the morphology, physiological and biochemical tests, and phylogenetic analysis, two isolated strains, WFC 414 and WFC 502, could be identified as *L. paracasei* and *L. plantarum*, respectively.

### Survival of *Lactiplantibacillus plantarum* and *Lacticaseibacillus paracasei* cells in simulated gastric and intestinal condition

**FIGURE 4:**
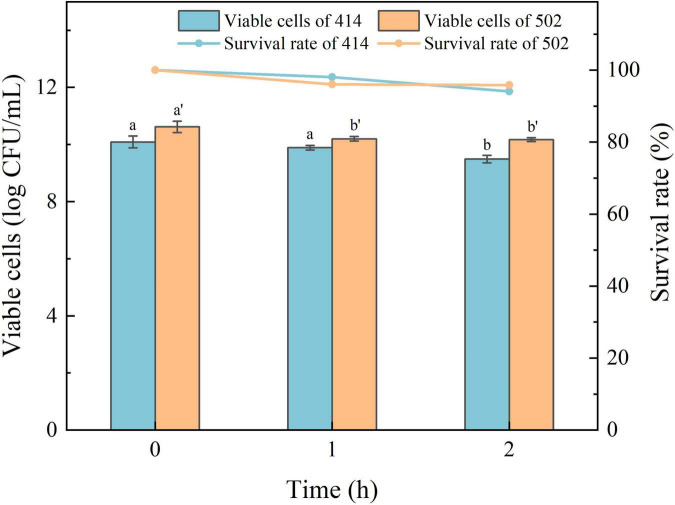
The survival rate of WFC 414 and WFC 502 cells incubated in simulated gastric juice for 1 and 2 h. Different letters (a, b or a’, b’) indicate significant differences at *p* < 0.05. Error bars show standard deviation.

**FIGURE 5:**
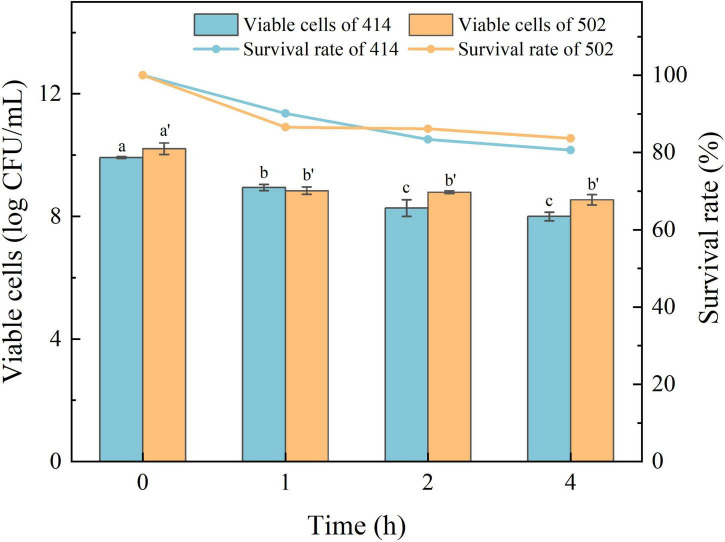
The survival rate of WFC 414 and WFC 502 cells incubated in simulated intestinal juice for 1, 2, and 4 h. Different letters (a, b, c or a’, b’) indicate significant differences at *p* < 0.05. Error bars show standard deviation.

The acid in gastric juice inhibits and kills most bacteria that enter the stomach. The ability to tolerate the environment in gastric juice and bile salts, and resist digestion by the enzymes are the characteristics required for LAB to survive in the human digestive system, colonize the intestinal tract, and perform their probiotic functions (34). The simulated gastric juice tolerance results of the two isolated strains are shown in Figure 4. The relative viability of both WFC 414 (92%) and WFC 502 (95%) was decreased after 2 h of exposure to SGJ at low pH (2.0) containing 0.3% pepsin. The viable cell count of WFC 414 showed a significant decrease in the second hour (*p* < 0.05). While the viable cell count of WFC 502 reduced significantly in the first hour (*p* < 0.05), the reduction was not significant in the second hour (*p* > 0.05). The different performance of the two strains in the simulated gastric juice could be due to the sensitivity to acidic conditions. The rapid decrease of WFC 502 in the first hour suggests that it is more sensitive to sudden changes in pH than WFC 414. When WFC 414 and WFC 502 were exposed to simulated intestinal juice containing 0.2% trypsin and 0.6% bile salts, the survival profiles over 4 h were recorded and shown in Figure 5. Incubated after 4 h in simulated intestinal juice, the relative viability of WFC 414 and WFC 502 was 80.7 and 83.6%, respectively. The viable cell count of WFC 414 decreased significantly and consistently during the first 3 h in simulated intestinal juice (*p* < 0.05). However, the number of viable cells only reduced in the first hour (*p* < 0.05) for WFC 502 and the change in the last 3 h was not significant (*p* > 0.05).

Some *Lactobacillus* are highly tolerant to only low pH conditions or bile salt conditions. However, survival was reduced under simulated gastrointestinal environments in the presence of pepsin or trypsin (35). This observation may be due to the destruction of bacterial cells by the existence of enzymes, leading them sensitive to low pH and bile salts (36). Several studies have used grain fibers or polysaccharides to immobilize and protect bacterial cells from adverse factors (37, 38). However, this may impact the taste of the juice products. So it is crucial to screen LAB resistant to gastrointestinal digestion by themselves to be useful as fermenters. Since WFC 414 and WFC 502 have a minimal reduction in viability after incubating in gastrointestinal conditions, they can be considered to have probiotic potential.

### Fermentation of the soluble sugar and organic acids content of apple juice by *Lactiplantibacillus plantarum* and *Lacticaseibacillus paracasei* cells

The sugar-acid ratio in apple juice can be altered by LAB metabolism to balance the flavor, chemical properties and stability of FAJ. Fructose, glucose, and sucrose are the predominant sugars in apple juice and can be utilized and consumed by *L. plantarum* and *L. paracasei*. The content of the three sugars in apple juice decreased during the fermentation of *L. paracasei* WFC 414 and *L. plantarum* WFC 502 (Table 3). The most significant decrease was observed in fructose and glucose. After 2 days of fermentation, the fructose in FAJ decreased by 5.40 and 4.94 mg/mL. WFC 502 no longer consumed fructose, while WFC 414 continued to reduce fructose by 1.81 mg/mL during the subsequent two days’ fermentation. Glucose maintained a decreasing trend over the 4 days of fermentation by WFC 414 and WFC 502 and eventually decreased by 7.09 (*p* < 0.05) and 7.47 mg/mL (*p* < 0.05). Many studies have reported that fructose and glucose are the primary energy sources for *Lactobacillus* strains (39, 40). Sucrose in apple juice was much lower than fructose and glucose, and its consumption was minimal. After four days of fermentation, sucrose decreased by 2.59 and 2.36 mg/mL. In contrast, *L. paracasei* WFC 414 may prefer to consume sugars in juice than *L. plantarum* WFC 502. A similar phenomenon was observed in the fermentation of blueberry juice using LAB by Jahandideh et al. (39). The metabolism of sugars by different LAB varies from strain to strain.

**TABLE 3:** Soluble sugars (fructose, glucose, and sucrose) and organic acids (malic acid, lactic acid, oxalic acid, pyruvic acid, citric acid, and tartaric acid) of apple juice before and after fermentation by WFC 414 and WFC 502.

	PAJ	FAJ			
	Blank	WFC 414		WFC 502	
Time (d)	0	2	4	2	4
Fructose (mg/mL)	91.52 ± 1.95^a^	86.12 ± 0.80^ab^	84.31 ± 4.37^b^	86.58 ± 0.78^ab^	86.76 ± 0.18^ab^
Glucose (mg/mL)	49.75 ± 2.87^a^	45.41 ± 1.47^ab^	42.66 ± 2.33^b^	43.71 ± 0.39^b^	42.28 ± 0.12^b^
Sucrose (mg/mL)	15.40 ± 1.22^a^	13.60 ± 0.37^b^	12.81 ± 0.69^b^	13.42 ± 0.23^b^	13.04 ± 0.05^b^
Malic acid (mg/mL)	2.44 ± 0.07^a^	0.05 ± 0.00^b^	0.05 ± 0.00^b^	0.03 ± 0.00^b^	0.06 ± 0.01^b^
Lactic acid (mg/mL)	ND	2.63 ± 0.02^c^	3.48 ± 0.12^b^	3.61 ± 0.12^b^	5.94 ± 0.10^a^
Oxalic acid (mg/mL)	0.27 ± 0.01^c^	0.27 ± 0.00^b^	0.26 ± 0.00^a^	0.27 ± 0.00^bc^	0.27 ± 0.00^bc^
Pyruvic acid (mg/mL)	0.01 ± 0.00^c^	0.01 ± 0.00^b^	0.02 ± 0.00^a^	0.01 ± 0.00^bc^	0.01 ± 0.00^bc^
Citric acid (mg/mL)	ND	ND	ND	ND	ND
Tartaric acid (mg/mL)	ND	ND	ND	ND	ND

Different letters (a, b, c) indicate significant differences at *p* < 0.05. ND, not detected.

Malic acid is one of the most important organic acids in apple juice. The conversion of malic acid to lactic acid is the sign of the fermentation process of LAB (41). As illustrated in Table 3, the malic acid concentration in unfermented PAJ was 2.44 ± 0.07 mg/mL, and lactic acid was not detected. In 2-day fermentation, significant malic acid consumption by WFC 414 and WFC 502 was observed, with rates as high as 98 and 99%. Meanwhile, with the catalysis of malolactic enzyme produced by WFC 414 and WFC 502, lactic acid began to be produced and increased to 2.63 ± 0.02 and 3.61 ± 0.12 mg/mL. During the following 2 days of fermentation, the malic acid content no longer decreased, while the lactic acid content continued to increase significantly to 3.48 ± 0.12 and 5.94 ± 0.10 mg/mL. This result indicates that LAB may have other metabolic pathways for carbon sources. At the same time, the amount of lactic acid content after fermentation had an effect on the juice aroma due to its characteristic odor. The higher amount of lactic acid in WFC 502 may gave it much fruity and grassy aroma than WFC 414 (Figure 1). The lesser amount of lactic acid in WFC 414 accentuated the sweet and floral aroma. A small quantity of pyruvate was detected in both PAJ and FAJ, and the change in its content was not significant (*p* > 0.05). Pyruvate is an intermediate product in the organism’s metabolic pathways, such as the tricarboxylic acid cycle and glycolysis. Pyruvate can be produced from glucose via the glycolytic pathway but can also be converted to lactic acid via the lactic acid fermentation pathway (42). Therefore, its content may show a dynamic equilibrium. The variation trend of oxalic acid content in FAJ is similar to that of pyruvate and was not significant (*p* > 0.05) as same as the change of oxalic acid during the fermentation of Sea buckthorn-apple juice (41). Citric acid and tartaric acid were not detected in the apple juice before and after fermentation. This result may be related to the different apple varieties. Citric acid is an intermediate of the tricarboxylic acid cycle. It is also in the dynamic of being consumed while being generated.

### Total polyphenols and individual phenolics content of apple juice by *Lactiplantibacillus plantarum* and *Lacticaseibacillus paracasei* cells

Apple polyphenols are bioactive substances that can improve hypertension, hyperlipidemia, hyperglycemia, and inflammatory response (43). The contents of total polyphenols and representative individual phenolics such as chlorogenic acid, gallic acid, procyanidin B_2_, epicatechin, rutin, catechin, protocatechuic acid, phloretin, and phlorizin in apple juice were determined (Table 4). The total phenolic content in apple juice decreased from 331.43 ± 3.38 to 286.52 ± 3.15 (*p* < 0.05) and 258.25 ± 5.66 mg GAE/L (*p* < 0.05) after fermentation by WFC 414 and WFC 502, respectively. This may be due to *Lactobacillus* synthesizes decarboxylase, tannase, and glycosidase during fermentation to enhance the decomposition and metabolic conversion of polyphenols (42). Gallic acid can improve the antioxidant properties of FAJ, and its significant increase may come from the hydrolysis of tannins in the juice. And the increase in phloretin content may be attributed to the release of phlorizin catalyzed by β-glucosidase (44). Chlorogenic acid and procyanidin B_2_ are the most abundant water-soluble polyphenols in apple juice. The fermentation of WFC 414 and WFC 502 reduced them less, probably due to the acidic environment provided by *Lactobacillus* and the anaerobic conditions during fermentation that slowed their oxidation. Catechin and epicatechin are the main polyphenols in apple juice. Fermentation reduced the concentration of epicatechin, but there was little change in catechin. The changes of polyphenols in apple juice before and after fermentation are closely related to apple varieties and fermentation strains. In general, although the total phenolic content decreases after fermentation, the monomeric phenols converted from macromolecular polyphenols have superior bioavailability. Thus, FAJ can exert a higher nutritional value in the human body.

**TABLE 4:** Individual phenolic compounds of apple juice before and after fermentation by WFC 414 and WFC 502.

Phenolics (mg/L)	PAJ	FAJ	
	Blank	WFC 414	WFC 502
Chlorogenic acid	125.34 ± 1.86^a^	114.60 ± 1.70^b^	116.59 ± 0.87^b^
Gallic acid	1.30 ± 0.10^c^	3.96 ± 0.05^a^	3.40 ± 0.11^b^
Procyanidin B_2_	51.74 ± 0.68^a^	47.41 ± 1.46^b^	46.00 ± 1.90^b^
Epicatechin	28.66 ± 1.19^a^	22.68 ± 0.62^b^	24.05 ± 0.44^b^
Rutin	1.81 ± 0.02^a^	1.77 ± 0.02^a^	1.76 ± 0.02^a^
Catechin	6.56 ± 0.05^ab^	6.49 ± 0.05^a^	6.66 ± 0.06^b^
Protocatechuic acid	6.22 ± 0.27^c^	9.07 ± 0.17^a^	8.46 ± 0.16^b^
Phlorizin	1.22 ± 0.06^a^	0.96 ± 0.08^b^	0.90 ± 0.05^b^
Phloretin	2.22 ± 0.09^b^	2.85 ± 0.12^a^	3.05 ± 0.17^a^

Different letters (a, b, c) indicate significant differences at *p* < 0.05.

### The volatile profiles of fermented apple juice by *Lactiplantibacillus plantarum* and *Lacticaseibacillus paracasei* cells

Volatile compounds in fruit juice may give consumers a pre-impression through its odor before tasting the juice. The pre-impression has a specific impact on the consumer’s evaluation of the products. Therefore, the change in the apple juice aroma by the WFC 414 and WFC 502 fermentations is also an indication of the comprehensive evaluation of strains. Fifty-nine volatile compounds contributing to apple juice aroma were detected in PAJ and FAJ by HS-SPME-GC–MS (Table 5). Among them are nineteen alcohols, twenty esters, seven aldehydes, three ketones, six acids, and four other terpenoids and phenols.

**TABLE 5:** The volatile compounds content in pasteurized apple juice (PAJ) and WFC 414 and WFC 502 fermented apple juice (FAJ).

Number	Compounds	Formula	CAS	Content/(μg/L)			Odor descriptor
				PAJ	WFC 414	WFC 502	
***Alcohols***							
1	Ethanol	C_2_H_6_O	64-17-5	3.28 ± 0.14	112.25 ± 5.63	8.18 ± 0.58	Sweet; strong alcoholic
2	2-Methyl-1-propanol	C_4_H_10_O	78-83-1	–	33.87 ± 0.70	4.15 ± 0.06	Winey; cortex
3	(*S*)-(+)-2-Pentanol	C_5_H_12_O	26184-62-3	–	1.74 ± 0.03	2.34 ± 0.32	Wild berry; astringent
4	2-Methyl-1-butanol	C_5_H_12_O	137-32-6	298.80 ± 0.62	462.12 ± 5.21	332.88 ± 18.52	Winey; cocoa; malt
5	1-Pentanol	C_5_H_12_O	71-41-0	–	11.65 ± 0.45	–	Sweet; balsam
6	3-Methyl-3-Buten-1-ol	C_5_H_10_O	763-32-6	–	–	1.49 ± 0.31	Sweet fruity
7	2-Methyl-2-Buten-1-ol	C_5_H_10_O	4675-87-0	–	3.01 ± 0.70	2.40 ± 0.53	Green; oily
8	1-Hexanol	C_6_H_14_O	111-27-3	259.19 ± 4.27	207.44 ± 9.88	316.92 ± 17.37	Fruity; sweet; floral
9	(*E*)-3-Hexen-1-ol	C_6_H_12_O	928-97-2	–	–	1.33 ± 0.07	Leafy; floral; moss
10	*Trans*-2-Hexenol	C_6_H_12_O	928-95-0	7.89 ± 0.06	–	19.17 ± 1.73	Leafy; walnut; fruity
11	1-Octen-3-ol	C_8_H_16_O	3391-86-4	–	2.68 ± 0.14	4.41 ± 0.82	Mushroom; earthy; chicken
12	6-Methyl-5-Hepten-2-ol	C_8_H_16_O	1569-60-4	–	72.64 ± 1.73	–	Sweet; coriander
13	Linalool	C_10_H_18_O	78-70-6	–	6.15 ± 0.09	–	Citrus; floral sweet; blueberry
14	1-Octanol	C_8_H_18_O	111-87-5	4.44 ± 0.31	1.36 ± 0.04	5.84 ± 1.25	Orange; rose; mushroom
15	9-Decen-1-ol	C_10_H_20_O	13019-22-2	6.94 ± 0.24	–	–	Dewy; rose; fresh
16	1-Nonanol	C_9_H_20_O	143-08-8	–	6.06 ± 0.08	7.35 ± 0.25	Fresh; floral; orange
17	Ipsdienol	C_10_H_16_O	35628-00-3	3.86 ± 0.17	13.12 ± 0.27	–	Pine balsamic
18	Geraniol	C_10_H_18_O	5944-20-7	–	49.10 ± 1.81	–	Rose
19	1,3-Octanediol	C_8_H_18_O_2_	23433-05-8	30.74 ± 1.45	21.02 ± 0.39	18.91 ± 0.18	Musty
	*Subtotal*			614.11 ± 7.50	1004.20 ± 2.81	725.37 ± 3.42	
***Esters***							
1	1,2-Ethylene glycol diacetate	C_6_H_10_O_4_	111-55-7	–	–	0.08 ± 0.00	Floral; ester; alcoholic
2	Ethyl butyrate	C_6_H_12_O_2_	105-54-4	31.61 ± 0.75	–	–	Apple; fruity; pineapple; cognac
3	Ethyl 2-methylbutyrate	C_7_H_14_O_2_	7452-79-1	26.58 ± 0.84	–	–	Sharp sweet; apple; fruity
4	Butyl acetate	C_6_H_12_O_2_	123-86-4	22.35 ± 0.17	7.43 ± 0.66	6.54 ± 0.47	Fruity; banana; pear
5	2-Methylbutyl acetate	C_7_H_14_O_2_	624-41-9	101.68 ± 1.26	–	–	Ripe fruit; sweet; banana; juicy
6	Heptyl acetate	C_9_H_18_O_2_	112-06-1	–	–	1.97 ± 0.05	Rum; ripe fruit; pear; apricot
7	Pentyl acetate	C_7_H_14_O_2_	628-63-7	4.03 ± 0.02	–	–	Fruity; pear; banana; apple
8	Butyl butyrate	C_8_H_16_O_2_	109-21-7	3.10 ± 0.02	–	–	Pineapple; cherry; ripe fruit
9	Butyl 2-methylbutanoate	C_9_H_18_O_2_	15706-73-7	7.88 ± 0.15	–	–	Fruity; cocoa; grassy
10	Hexyl acetate	C_8_H_16_O_2_	142-92-7	22.57 ± 1.67	2.21 ± 0.20	–	Fruity; apple; sweet
11	Pentyl hexanoate	C_11_H_22_O_2_	540-07-8	4.10 ± 0.11	–	–	Sweet; fruity; apple
12	Hexyl 2-methylbutyrate	C_11_H_22_O_2_	10032-15-2	6.77 ± 0.13	–	–	Fruity; apple; spicy
13	Methyl 2-hydroxy-3-methyl pentanoate	C_7_H_14_O_3_	41654-19-7	–	1.46 ± 0.04	1.21 ± 0.02	Fruity; ester; caramellic
14	Ethyl 3-hydroxybutyrate	C_6_H_12_O_3_	5405-41-4	6.84 ± 0.05	5.76 ± 0.23	5.55 ± 0.48	Fruity; apple-skin
15	Nonyl acetate	C_11_H_22_O_2_	143-13-5	6.91 ± 0.02	–	–	Fruity; sweet
16	Ethyl 3-hydroxyhexanoate	C_8_H_16_O_3_	2305-25-1	–	5.75 ± 0.85	–	Fruity; woody; spicy
17	2-Phenylethyl Acetate	C_10_H_12_O_2_	103-45-7	8.23 ± 1.89	–	–	Rose, honey, tobacco
18	Geranyl isovalerate	C_15_H_26_O_2_	109-20-6	–	5.07 ± 0.45	–	Green; fruity; apple; rose
19	Nerolidyl acetate	C_17_H_28_O_2_	2306-78-7	–	–	3.54 ± 1.29	Sweet; citrus; waxy; woody
20	Methyl palmitate	C_17_H_34_O_2_	112-39-0	–	1.30 ± 0.01	–	Waxy; orris
	*Subtotal*			252.66 ± 3.49	28.97 ± 1.86	18.89 ± 1.29	
***Aldehydes***							
1	Hexanal	C_6_H_12_O	66-25-1	43.07 ± 0.43	–	–	Fresh; grassy; fruity
2	(*E*)-2-Hexenal	C_6_H_10_O	6728-26-3	75.64 ± 1.03	–	33.69 ± 0.07	Apple; banana; cheesy
3	Octanal	C_8_H_16_O	124-13-0	2.86 ± 0.14	–	–	Lemon; waxy; citrus; grassy
4	Nonanal	C_9_H_18_O_2_	124-19-6	7.30 ± 0.75	–	2.48 ± 0.36	Waxy; rose; orris; peely
5	Benzaldehyde	C_7_H_6_O	100-52-7	9.84 ± 0.42	3.46 ± 0.34	3.18 ± 0.22	Sweet; almond; cherry
6	2,4-dimethyl benzaldehyde	C_9_H_10_O	15764-16-6	23.19 ± 1.10	19.14 ± 0.20	17.54 ± 0.94	Cherry; almond; vanilla
7	Tetradecanal	C_14_H_28_O	124-25-4	4.53 ± 0.43	–	–	Flower; waxy; amber; musk
	*Subtotal*			166.42 ± 4.10	22.60 ± 0.15	56.89 ± 0.89	
***Ketones***							
1	Damascenone	C_13_H_18_O	23696-85-7	6.73 ± 0.36	14.92 ± 1.02	12.18 ± 0.02	Sweet; fruity; rose
2	3-Octanone	C_8_H_16_O	106-68-3	–	–	0.75 ± 0.10	Fresh; herbal; lavender; sweet
3	Acetoin	C_4_H_8_O_2_	513-86-0	–	12.04 ± 0.53	12.18 ± 0.03	Sweet; creamy; fatty
	*Subtotal*			6.73 ± 0.36	26.96 ± 1.55	25.12 ± 0.16	
***Acid***							
1	Butanoic acid	C_4_H_8_O_2_	107-92-6	–	14.62 ± 0.28	–	Cheesy; fruit rancid; sweaty
2	2-Methyl-butanoic acid	C_5_H_10_O_2_	116-53-0	32.73 ± 0.53	40.94 ± 0.86	40.34 ± 2.87	Pungent acid; cheesy; sweaty
3	3-Methyl-pentanoic acid	C_6_H_12_O_2_	105-43-1	5.71 ± 0.10	15.66 ± 0.99	15.83 ± 0.57	Sharp acidic; cheesy; fruity
4	Hexanoic acid	C_6_H_12_O_2_	142-62-1	–	26.89 ± 2.77	24.25 ± 0.85	Sour; fatty; cheesy
5	Octanoic acid	C_8_H_16_O_2_	124-07-2	–	3.56 ± 0.02	0.55 ± 0.03	Cheesy; waxy; vegetable
6	Nonanoic acid	C_9_H_18_O_2_	112-05-0	5.19 ± 0.17	2.48 ± 0.25	3.91 ± 0.15	Waxy; cheesy; cultured dairy
	*Subtotal*			43.63 ± 0.77	104.15 ± 2.54	84.86 ± 2.73	
***Others***							
1	α-Farnesene	C_15_H_24_	502-61-4	48.04 ± 0.89	28.55 ± 1.05	32.50 ± 1.28	Citrus; herbal; lavender
2	Phenol	C_6_H_6_O	108-95-2	128.27 ± 2.45	–	–	Plastic; rubber
3	*Trans*-Linalool oxide (furanoid)	C_10_H_18_O_2_	34995-77-2	–	4.59 ± 0.16	–	Floral
4	Eugenol	C_10_H_12_O_2_	97-53-0	–	–	5.41 ± 0.05	Sweet; clove; honey
	*Subtotal*			176.31 ± 3.35	33.14 ± 0.90	37.92 ± 1.33	

Each data in the table is represented by the mean ± standard deviation (n = 3).

The symbol “–” means that the substance was not detected.

Alcohol is the most abundant aroma component in PAJ and FAJ. Alcohols bring an advanced aroma to fruit juices and can act as excellent solvents for other aromatic compounds, contributing significantly to the retention of their aroma (45). The types of alcohol in PAJ are not many, but they account for 48.70% of the total volatile compounds content. 2-Methyl-1-butanol has winey and malt odors, and is derived from isoleucine (46). The 1-hexanol in juice may contribute to fruity and floral aromas. 2-Methyl-1-butanol and 1-hexanol were significantly higher in PAJ than other alcohols and also increased to varying degrees in FAJ after fermentation. It may be considered the key alcohol in apple juice. Besides, *L. paracasei* WFC 414 fermentation produced a large amount of ethanol (108.97 μg/L), 6-methyl-5-hepten-2-ol (72.64 μg/L), and geraniol (49.10 μg/L). *L. paracasei* may convert glucose and amino acids into ethanol, producing a more pronounced sweet aroma in juice (15). The presence of geraniol and linalool enables the FAJ to exhibit rose and citrus aromas. Similar to what Han et al. (47) had reported, *L. plantarum* WFC 502 fermentation increased the content of trans-2-hexenol (11.28 μg/L) with leafy, walnut, and fruity aromas. 2-Methyl-1-propanol, (S)-(+)-2-pentanol, 2-methyl-2-buten-1-ol, 1-octen-3-ol, and 1-nonanol were produced in both FAJs, and they come in fragrances like a winey, wild berry, green, mushroom, fresh and orange. Notably, 1,3-octanediol is described as having a musty odor. Fermentation of both strains significantly reduced the content of this substance in apple juice. So, *Lactobacillus* fermentation promoted the production of alcohols and enriched the background aroma of FAJ.

Esters are a class of volatile compounds with the greatest contribution to apple juice aroma and are the primary source of fruity aromas. Thirteen esters were detected in PAJ, and the most dominant of which was 2-methylbutyl acetate, the characteristic substance in fresh apple juice aroma. 2-methylbutyl acetate imparts fruity and sweet aromas to the juice (48). Other prominent esters in PAJ, such as ethyl butyrate, ethyl 2-methylbutyrate, hexyl acetate, nonyl acetate, and 2-phenylethyl acetate, were almost completely consumed during the fermentation of *L. paracasei* and *L. plantarum*. The reduction in esters during juice fermentation is related to the volatility of the esters. It may also be attributed to the high esterase activity of *Lactobacillus*, which promotes the degradation of esters (49). Ester reduction was also observed in LAB fermented star fruit juice and cashew juice (26, 49). In addition to methyl 2-hydroxy-3-methyl pentanoate (fruity and caramellic), three new esters, ethyl 3-hydroxyhexanoate (fruity), geranyl isovalerate (fruity and rose aroma), and methyl palmitate (waxy and orris) were also produced during WFC 414 fermentation. WFC 502 fermentation also added three new esters, 1,2-ethylene glycol diacetate (floral and alcoholic), heptyl acetate (rum and pear aroma) and nerolidyl acetate (sweet and citrus). Changes in esters during malolactic fermentation are strain-specific (50). *Lactobacillus* fermentation largely altered the alcohols and esters in the juice, promoting significant changes in the volatility profile.

Fermented apple juice contains more volatile acids than PAJ. Acids are mainly produced during fermentation and tend to release odors that be not readily accepted by the public (51). For example, 2-methyl-butanoic acid, 3-methyl-pentanoic acid, hexanoic acid and octanoic acid, acids presented in FAJ, all exhibited sharp acidic, cheesy, waxy, sweaty and other undesirable odors to varying degrees (52). However, they also endowed FAJ with the characteristic microbial fermentation aroma. Butanoic acid (cheesy, fruit rancid and sweaty) was only found in the FAJ fermented by *L. paracasei*.

Fermentation essentially reduced the total amount of aldehydes. Aldehyde in juice is unstable because it is easily degraded by microorganisms through redox to produce corresponding alcohol or acid (45). Degradation of C6 aldehydes such as hexanal and (*E*)-2-hexenal may promote the increase of C6 alcohols and C6 acids such as 1-hexanol, *trans*-2-hexenol, and hexanoic acid. Only one ketone, damascenone (sweet, fruity, and rose), was detected in PAJ. The content of damascenone in FAJ was increased, and acetoin and a small amount of 3-octanone were newly added. Acetoin has a sweet and creamy odor, probably from citric acid metabolism (53). In addition, several terpenoids and phenolics were detected. Fermentation of both strains reduced α-farnesene (citrus and herbal) and completely removed phenol with unpleasant odors of plastic and rubber. WFC 414 produced *trans*-linalool oxide (furanoid) to enhance the floral aroma of FAJ. In contrast, WFC502 produced eugenol to give FAJ a clove and honey aroma. Many volatile compounds influence the aroma characteristics and quality of apple juice in fermentation, even though some have low concentration thresholds. LAB fermentation was suggested to be an effective way to enrich the aroma of FAJ.

## Conclusion

Two new strains of *Lacticaseibacillus paracasei* (WFC 414) and *Lactiplantibacillus plantarum* (WFC 502) isolated, screened and identified from pickles were applied to apple juice fermentation. Their growth viability and fermentation capacity in apple juice, as well as their gastrointestinal activity under simulated conditions, were all satisfactory. Through fermentation, WFC 414 and WFC 502 reduced soluble sugars and total polyphenols in apple juice, fully used malic acid to produce lactic acid. In addition, the fermentation of the strains regulated the volatile aroma components of apple juice. Both strains up-regulated the sweet, fruity, floral, and unique fermentation odors from alcohols, ketones, and acids. Overall, the two strains of *Lactobacillus* screened may have broad application prospects in apple juice fermentation and contribute to developing new nutritional apple juice probiotic products.

## Data availability statement

The data presented in this study are deposited in the NCBI database (https://www.ncbi.nlm.nih.gov/), accession numbers: OP735580 and OP735581.

## Author contributions

JL: writing—original draft, data curation, investigation, methodology, formal analysis, resources, software, and visualization. HD: data curation, investigation, methodology, formal analysis, resources, software, and visualization. CH: writing—review and editing and supervision. PZ: supervision. YM: writing—review and editing, validation, resources, supervision, and project administration. All authors contributed to the article and approved the submitted version.

## Footnotes

1
http://www.ncbi.nlm.nih.gov

## References

[1] Ivey M Massel M Phister TG. Microbial interactions in food fermentations. *Ann Rev Food Sci Technol.* (2013) 4:141–62. 10.1146/annurev-food-022811-101219 23190140

[2] Gammacurta M Marchand S Albertin W Moine V Revel GD. Impact of yeast strain on ester levels and fruity aroma persistence during aging of bordeaux red wines. *J Agric Food Chem.* (2014) 62:5378–89. 10.1021/jf500707e 24871631

[3] Xu X Bao Y Wu B Lao F Hu X Wu J. Chemical analysis and flavor properties of blended orange, carrot, apple and Chinese jujube juice fermented by selenium-enriched probiotics. *Food Chem.* (2019) 289:250–8. 10.1016/j.foodchem.2019.03.068 30955609

[4] Cagno RD Coda R Angelis M Gobbetti M. Exploitation of vegetables and fruits through lactic acid fermentation. *Food Microbiol.* (2013) 33:1–10. 10.1016/j.fm.2012.09.003 23122495

[5] Dagfinn A Edward G Paolo B Fadnes LT Nana K Teresa N Fruit and vegetable intake and the risk of cardiovascular disease, total cancer and all-cause mortality-a systematic review and dose-response meta-analysis of prospective studies. *Int J Epidemiol.* (2017) 46:1029–56. 10.1093/ije/dyw319 28338764 PMC5837313

[6] Bavisetty SCB Venkatachalam K. Physicochemical qualities and antioxidant properties of juice extracted from ripe and overripe wax apple as affected by pasteurization and sonication. *J Food Process Preserv.* (2021) 45:e15524. 10.1111/jfpp.15524

[7] Xiao YS Xiong T Peng Z Liu CG Huang T Yu H Correlation between microbiota and flavours in fermentation of Chinese Sichuan Paocai. *Food Res Int.* (2018) 114:123–32. 10.1016/j.foodres.2018.06.051 30361008

[8] Zheng J Wittouck S Salvetti E Franz CMAP Harris HMB Mattarelli P A taxonomic note on the genus *Lactobacillus*: Description of 23 novel genera, emended description of the genus *Lactobacillus* Beijerinck 1901, and union of Lactobacillaceae and Leuconostocaceae. *Syst Evol Microbiol.* (2020) 70:2782–858. 10.1099/ijsem.0.004107 32293557

[9] Zhongxi LI Jing TENG Yilu LYU Xiaoqian HU Zhao Y. Enhanced antioxidant activity for apple juice fermented with *Lactobacillus plantarum* ATCC14917. *Molecules.* (2018) 24:51. 10.3390/molecules24010051 30586844 PMC6337214

[10] Bai L Ruxianguli M Wang L. Effects of four individual lactic acid bacteria on the physical and chemical and antioxidant properties of Kuqa apple juice during fermentation. *J Food Process Preserv.* (2021) 45:e15385. 10.1111/jfpp.15385

[11] Li Z Teng J Lyu Y Hu X Zhao Y Wang M. Enhanced antioxidant activity for apple juice fermented with *Lactobacillus plantarum* ATCC14917. *Molecules.* (2018) 24:51. 10.3390/molecules24010051 PMC6337214 30586844

[12] Kaprasob R Kerdchoechuen O Laohakunjit N Sarkar D Shetty K. Fermentation-based biotransformation of bioactive phenolics and volatile compounds from cashew apple juice by select lactic acid bacteria. *Process Biochem.* (2017) 59:141–9. 10.1016/j.procbio.2017.05.019

[13] Wu CY Li TL Qi J Jiang T Xu HD Lei HJ. Effects of lactic acid fermentation-based biotransformation on phenolic profiles, antioxidant capacity and flavor volatiles of apple juice. *LWT-Food Sci Technol.* (2020) 122:109064. 10.1016/j.lwt.2020.109064

[14] Park S Son HK Chang HC Lee JJ. Effects of cabbage-apple juice fermented by *Lactobacillus plantarum* EM on lipid profile improvement and obesity amelioration in rats. *Nutrients.* (2020) 12:1135. 10.3390/nu12041135 32325640 PMC7230889

[15] Han M Zhang M Wang X Bai X Gao Z. Cloudy apple juice fermented by *Lactobacillus* prevents obesity via modulating gut microbiota and protecting intestinal tract health. *Nutrients.* (2021) 13:971. 10.3390/nu13030971 33802755 PMC8002442

[16] Wastyk HC Fragiadakis GK Perelman D Dahan D Merrill BD Yu FQB Gut-microbiota-targeted diets modulate human immune status. *Cell.* (2021) 184:4137–53. 10.1016/j.cell.2021.06.019 34256014 PMC9020749

[17] Gille D Schmid A Walther B VERGèRES G. Fermented food and non-communicable chronic diseases: a review. *Nutrients.* (2018) 10:448. 10.3390/nu10040448 29617330 PMC5946233

[18] Zhao Y Wang P Zhan P Tian HL Lu C Tian P. Aroma characteristics of cloudy kiwifruit juices treated with high hydrostatic pressure and representative thermal processes. *Food Res Int.* (2021) 139:109841. 10.1016/j.foodres.2020.109841 33509465

[19] Batdorj B Trinetta V Dalgalarrondo M PRéVOST H Dousset X Ivanova I Isolation, taxonomic identification and hydrogen peroxide production by *Lactobacillus delbrueckii* subsp. lactis T31, isolated from Mongolian yoghurt: inhibitory activity on food-borne pathogens. *J Appl Microbiol.* (2010) 103:584–93. 10.1111/j.1365-2672.2007.03279.x 17714391

[20] Moayyedi M Eskandari MH Rad A Ziaee E Khodaparast M Golmakani MT. Effect of drying methods (electrospraying, freeze drying and spray drying) on survival and viability of microencapsulated *Lactobacillus rhamnosus* ATCC 7469. *J Funct Foods.* (2018) 40:391–9. 10.1016/j.jff.2017.11.016

[21] Li H Huang J Wang Y Wang X Ren Y Yue T Study on the nutritional characteristics and antioxidant activity of dealcoholized sequentially fermented apple juice with *Saccharomyces cerevisiae* and *Lactobacillus plantarum* fermentation. *Food Chem.* (2021) 363:130351. 10.1016/j.foodchem.2021.130351 34147897

[22] Tian Y Gou XJ Niu PF Sun LJ Guo YR. Multivariate data analysis of the physicochemical and phenolic properties of not from concentrate apple juices to explore the alternative cultivars in juice production. *Food Anal Methods.* (2018) 11:1735–47. 10.1007/s12161-018-1169-2

[23] Yang Y Zhao PT Wang XY Cui GX Guo YR. Using a red-fleshed and six varieties of thinned young apple to make juice and their phytochemicals characterization. *J Food Process Preserv.* (2021) 45:e15361. 10.1111/jfpp.15361

[24] Filannino P Azzi L Cavoski I Vincentini O Rizzello CG Gobbetti M Exploitation of the health-promoting and sensory properties of organic pomegranate (*Punica granatum* L.) juice through lactic acid fermentation. *Int J Food Microbiol.* (2013) 163:184–92. 10.1016/j.ijfoodmicro.2013.03.002 23562695

[25] Dysvik A Rosa S Buffetto F Liland KH Westereng B. Secondary lactic acid bacteria fermentation with wood-derived xylooligosaccharides as a tool to expedite sour beer production. *J Agric Food Chem.* (2019) 68:301–14. 10.1021/acs.jafc.9b05459 31820631

[26] Alves Filho EDG Soares Rodrigues TH Narcisofernandes FA Fernandes Pereira AL Narain N Brito ED Chemometric evaluation of the volatile profile of probiotic melon and probiotic cashew juice. *Food Res Int.* (2017) 99:461–8. 10.1016/j.foodres.2017.05.030 28784506

[27] Ricci A Cirlini M Maoloni A Del Rio D Calani L Bernini V Use of dairy and plant-derived lactobacilli as starters for cherry juice fermentation. *Nutrients.* (2019) 11:213. 10.3390/nu11020213 30678152 PMC6412669

[28] Huang CH Li SW Huang L Watanabe K. Identification and Classification for the *Lactobacillus casei* Group. *Front Microbiol.* (2018) 9:1974. 10.3389/fmicb.2018.01974 30186277 PMC6113361

[29] Kunene NF Geornaras I Von Holy A Hastings JW. Characterization and determination of origin of lactic acid bacteria from a sorghum-based fermented weaning food by analysis of soluble proteins and amplified fragment length polymorphism fingerprinting. *Appl Environ Microbiol.* (2000) 66:1084–92. 10.1128/AEM.66.3.1084-1092.2000 10698775 PMC91946

[30] Tanguler H Erten H. Occurrence and growth of lactic acid bacteria species during the fermentation of shalgam (salgam), a traditional Turkish fermented beverage. *LWT-Food Sci Technol.* (2012) 46:36–41. 10.1016/j.lwt.2011.10.026

[31] Kearney N Meng XC Stanton C Kelly J Fitzgerald GF Ross RP. Development of a spray dried probiotic yoghurt containing *Lactobacillus paracasei* NFBC 338. *Int Dairy J.* (2009) 19:684–9. 10.1016/j.idairyj.2009.05.003

[32] Randazzo CL Russo N Pino A Mazzaglia A Ferrante M Conti GO Effects of selected bacterial cultures on safety and sensory traits of Nocellara Etnea olives produced at large factory scale. *Food Chem Toxicol.* (2018) 115:491–8. 10.1016/j.fct.2018.03.045 29625158

[33] Slattery C Cotter PD O’TOOLE PW. Analysis of health benefits conferred by *Lactobacillus* Species from Kefir. *Nutrients.* (2019) 11:1252. 10.3390/nu11061252 31159409 PMC6627492

[34] Fang SH Lai YJ Chou CC. The susceptibility of *Streptococcus thermophilus* 14085 to organic acid, simulated gastric juice, bile salt and disinfectant as influenced by cold shock treatment. *Food Microbiol.* (2013) 33:55–60. 10.1016/j.fm.2012.08.012 23122501

[35] Tokatli M Gulgor G Elmaci SB Isleyen NA Ozcelik F. In vitro properties of potential probiotic indigenous lactic acid bacteria originating from traditional pickles. *Biomed Res Int.* (2015) 2015:315819. 10.1155/2015/315819 26101771 PMC4460932

[36] Leandro ED Ginani VC De Alencar ER Pereira OG Rose ECP Do Vale HMM Isolation, identification, and screening of lactic acid bacteria with probiotic potential in silage of different species of forage plants, cocoa beans, and artisanal salami. *Probiotics Antimicrob Proteins.* (2021) 13:173–86. 10.1007/s12602-020-09679-y 32601953

[37] Blaiotta G La Gatta B Di Capua M Di Luccia A Coppola R Aponte M. Effect of chestnut extract and chestnut fiber on viability of potential probiotic Lactobacillus strains under gastrointestinal tract conditions. *Food Microbiol.* (2013) 36:161–9. 10.1016/j.fm.2013.05.002 24010594

[38] Shu GW He YX Chen L Song YJ Cao JL Chen H. Effect of xanthan-chitosan microencapsulation on the survival of *Lactobacillus acidophilus* in simulated gastrointestinal fluid and dairy beverage. *Polymers.* (2018) 10:588. 10.3390/polym10060588 30966622 PMC6403948

[39] Jahandideh F Mousavi SM Razavi SH. Utilization of *Echium amoenum* extract as a growth medium for the production of organic acids by selected lactic acid bacteria. *Food Bioprocess Technol.* (2012) 5:2275–9. 10.1007/s11947-011-0564-0

[40] Hashemi SMB Khaneghah AM Barba FJ Nemati Z Shokofti SS Alizadeh F. Fermented sweet lemon juice (*Citrus limetta*) using *Lactobacillus plantarum* LS5: Chemical composition, antioxidant and antibacterial activities. *J Funct Foods.* (2017) 38:409–14. 10.1016/j.jff.2017.09.040

[41] Tkacz K Chmielewska J Turkiewicz IP Nowicka P Wojdylo A. Dynamics of changes in organic acids, sugars and phenolic compounds and antioxidant activity of sea buckthorn and sea buckthorn-apple juices during malolactic fermentation. *Food Chem.* (2020) 332:127382. 10.1016/j.foodchem.2020.127382 32619943

[42] Yang J Sun Y Gao T Wu Y Tao Y. Fermentation and storage characteristics of “Fuji” apple juice using *Lactobacillus acidophilus*, *Lactobacillus casei* and *Lactobacillus plantarum*: microbial growth, metabolism of bioactives and in vitro bioactivities. *Front Nutr.* (2022) 9:833906. 10.3389/fnut.2022.833906 35223961 PMC8864132

[43] Zhang SQ Hu CY Wang XY Meng YH Guo YR. Polyphenols in fermented apple juice: Beneficial effects on human health. *J Funct Foods.* (2021) 76:104294. 10.1016/j.jff.2020.104294

[44] Avila M Hidalgo M Sanchez-Moreno C Pelaez C Requena T De Pascual-Teresa S. Bioconversion of anthocyanin glycosides by Bifidobacteria and *Lactobacillus*. *Food Res Int.* (2009) 42:1453–61. 10.1016/j.foodres.2009.07.026

[45] Chen HL Xiao GS Xu YJ Yu YS Wu JJ Zou B. High hydrostatic pressure and co-fermentation by *Lactobacillus rhamnosus* and *Gluconacetobacter xylinus* improve flavor of yacon-litchi-longan juice. *Foods.* (2019) 8:308. 10.3390/foods8080308 31374950 PMC6722649

[46] Chen C Lu YQ Yu HY Chen ZY Tian HX. Influence of 4 lactic acid bacteria on the flavor profile of fermented apple juiceInfluence of 4 lactic acid bacteria. *Food Biosci.* (2019) 27:30–6. 10.1016/j.fbio.2018.11.006

[47] Han M Wang X Zhang M Ren Y Yue T Gao Z. Effect of mixed Lactobacillus on the physicochemical properties of cloudy apple juice with the addition of polyphenols-concentrated solution. *Food Biosci.* (2021) 41:448. 10.1016/j.fbio.2021.101049

[48] Wang LY Zhang HX Lei HJ. Phenolics profile, antioxidant activity and flavor volatiles of pear juice: Influence of lactic acid fermentation using three *Lactobacillus* strains in monoculture and binary mixture. *Foods.* (2022) 11:11. 10.3390/foods11010011 35010138 PMC8750113

[49] Lu YY Tan CW Chen D Liu SQ. Potential of three probiotic lactobacilli in transforming star fruit juice into functional beverages. *Food Sci Nutr.* (2018) 6:2141–50. 10.1002/fsn3.775 30510715 PMC6261227

[50] Sumby KM Grbin PR Jiranek V. Microbial modulation of aromatic esters in wine: Current knowledge and future prospects. *Food Chem.* (2010) 121:1–16. 10.1016/j.foodchem.2009.12.004

[51] Wei JP Zhang YX Qiu Y Guo H Ju HM Wang YW Chemical composition, sensorial properties, and aroma-active compounds of ciders fermented with *Hanseniaspora osmophila* and *Torulaspora quercuum* in co- and sequential fermentations. *Food Chem.* (2020) 306:125623. 10.1016/j.foodchem.2019.125623 31606633

[52] Kaprasob R Kerdchoechuen O Laohakunjit N Thumthanaruk B Shetty K. Changes in physico-chemical, astringency, volatile compounds and antioxidant activity of fresh and concentrated cashew apple juice fermented with *Lactobacillus plantarum*. *J Food Sci Technology-Mysore.* (2018) 55:3979–90. 10.1007/s13197-018-3323-7 30228396 PMC6133829

[53] Hugenholtz J. Citrate metabolism in lactic acid bacteria. *FEMS Microbiol Rev.* (1993) 12:165–78. 10.1111/j.1574-6976.1993.tb00017.x

